# Cardiac FGF23 Increases Intracellular Calcium in Atrial Myocytes and the Susceptibility to Atrial Fibrillation Decreased in FGF23^f^ ^/^ ^f^MyHC^Cre^ ^/+^ Mice

**DOI:** 10.1111/jcmm.70517

**Published:** 2025-03-24

**Authors:** Xiao‐Qian Li, Mei‐Qiong Wu, Li‐Hua Fang, Qian Chen, Zhi‐Jie Chen, Zhu‐Hui Lin, Jian‐Quan Chen, Panashe Makota, Yang Li, Jian‐Cheng Zhang

**Affiliations:** ^1^ Shengli Clinical Medicine College of Fujian Medical University Fuzhou Fujian China; ^2^ Department of Cardiology Fujian Provincial Hospital, Fuzhou University Affiliated Provincial Hospital Fuzhou Fujian China; ^3^ Department of Cardiovascular Medicine Fuzhou First Hospital Affiliated With Fujian Medical University Fuzhou Fujian China; ^4^ Department of Critical Care Medicine Division Four Fujian Provincial Hospital, Fuzhou University Affiliated Provincial Hospital Fuzhou Fujian China; ^5^ Department of Cardiology The Sixth Medical Center, Chinese People's Liberation Army Hospital Beijing China

**Keywords:** atrial fibrillation, FGF23, intracellular calcium, RyR2

## Abstract

Exogenous fibroblast growth factor (FGF) 23 is closely associated with atrial fibrillation (AF) and is able to alter the cardiac electrophysiological activity by increasing intracellular calcium. While its arrhythmogenic mechanism remains unclear, this study aims to investigate the electrophysiological effects of cardiac FGF23 on intracellular calcium in atrial cells and its underlying mechanism. The incidence of AF was significantly decreased in FGF23^f/f^MyHC^Cre/+^ mice compared to Cre mice. A significant increase in the incidence of triggering activity (TA), L‐type calcium currents (I_Ca,L_) and systolic calcium transient was induced in neonatal mice atrial myocytes (NMAMs) from the overexpression of FGF23. Conversely, the opposite effects were exhibited as a reduced diastolic spontaneous calcium leak and weakened Na^+^/Ca^2+^ exchange (NCX) function in cardiac myocytes from FGF23^f/f^MyHCCre^/+^ mice, which can reduce incidences of AF induced by delayed after depolarization (DAD). In addition, ryanodine‐receptor 2 (RyR2) of calcium regulatory proteins was significantly downregulated in FGF23^f/f^MyHC^Cre/+^ mice and upregulated in FGF23 overexpression of NMAMs. In conclusion, overexpression of cardiac FGF23 may increase the susceptibility to AF due to DAD or TA induced by increasing intracellular calcium in atrial myocytes.

## 1 Introduction

Atrial fibrillation (AF) is the most common type of tachyarrhythmia, which shows increasing incidence and prevalence with the aging population. Based on the Global Health Data Exchange (GHDE), there were 37.574 million AF patients worldwide in 2017 (0.51% of the global population), exhibiting a prevalence increase of 33% compared with 20 years ago [1]. In a community cohort study of 50,684 patients, AF showed an annual 3% increase in its incidence from 2006 to 2018 [2]. AF can lead to cardiac dysfunction on the one hand, and a 5‐fold risk of stroke compared with the normal population on the other [3]. Its corresponding consequence lies in disability and high mortality, posing a serious burden on human health and the social economy. It is currently believed that AF will occur under the condition of trigger factors, and its maintenance depends on the atrial matrix. On the one hand, an ectopic excitatory focus is only a trigger factor for AF. On the other hand, atrial dilation, fibrosis and ultrastructural changes caused by various pathophysiological reasons are crucial for AF, such as myolysis, glycogen accumulation, changes in nuclear chromatin, mitochondria and the sarcoplasmic reticulum [4, 5]. These two factors jointly alter the electrical and structural remodelling of the atrium, thereby promoting the occurrence and maintenance of AF [6]. Recent years have seen great advances in the minimally invasive or interventional treatment of AF. However, partial cases of AF exhibit a high recurrence rate and lack effective treatment approaches, which can be attributed to the indistinct pathophysiological mechanism underlying the occurrence and maintenance of AF. The discovery of key molecules involved in atrial remodelling is of great significance for revealing the pathogenesis, early diagnosis and targeted intervention of AF.

FGF23, a newly discovered member of the endocrine FGF family, is predominantly secreted by osteocytes. Numerous cohort studies have suggested that the increased level of FGF23 is correlated with the occurrence of AF [7]. A study quantitatively measured more than 40 cardiovascular biomarkers in 638 patients and found that FGF23 was second only to the classical biomarker B brain natriuretic peptide (BNP), establishing it as a predictive biological marker for AF [8]. Several researchers have proposed that FGF23 may be a new key among indicators that can induce AF [9]. Understanding the role of the key molecule FGF23 may reveal additional causes of AF, which hold significant implications for the early prediction and elucidation of AF mechanisms.

The generation of AF requires rapid ectopic triggering involving foci and AF matrix. Abnormal intracellular calcium disposal figures prominently in the formation of ectopic rhythm during AF [10, 11, 12]. Intracellular calcium overload during the diastolic period is the main reason that facilitates the occurrence of DAD and causes ectopic rhythm [13]. Increased calcium inflow and Sarco/ER Ca^2+^‐ATPase (SERCA) activity in the SR can increase the calcium load of the SR. Calcium ion leakage during diastole may account for increased frequency of RyR2 opening caused by higher SR load, elevated expression and activity of RyR2 protein [9] as well as excessive activation of RyR2 channels due to phosphorylation induced by protein kinase A (PKA) [10] and calmodulin‐dependent protein kinase II (CAMKII) [14]. In addition, the reduced interaction between RyR2 and its stable subunits also modulates the likelihood of RyR2 opening [15, 16]. All of the above factors can lead to intracellular calcium overload and activate instantaneous transient inward current (I_ti_), thereby promoting the occurrence of DAD. TA will be induced at a certain threshold, generating ectopic rhythm and promoting the occurrence of AF.

FGF23 can increase intracellular calcium levels in isolated cardiomyocytes, as well as the contractility of primary mouse cardiomyocytes and ventricular muscle bundles [17]. FGF23 may cause intracellular calcium overload by directly or indirectly regulating the expression and function of calcium‐regulated proteins, leading to increased ectopic rhythm. From this point, FGF23 exerts an important role in the occurrence and maintenance of AF. Traditionally, it is believed that external stimulation causes the bone to release FGF23 into the circulation, thus resulting in cardiotoxic effects. Recent studies have indicated that the expression of FGF23 is not limited to bone, but can also be expressed in cardiomyocytes and up‐regulated in pathological cardiac remodelling and other environments [18, 19], suggesting that the cardiotoxicity of FGF23 may be partially due to the paracrine or autocrine effects of cardiac‐derived FGF23. The regulatory effect of cardiac FGF23 on intracellular calcium remains unclear.

In the present study, we generated a mouse model with cardiac‐specific knockout of FGF23 to elucidate the effect of the cardiac FGF23 itself on the progression of AF. Meanwhile, we isolated NMAMs overexpressing FGF23 via lentivirus gene transfer to investigate the electrophysiological effects of cardiac FGF23 on intracellular calcium in atrial cells and identify its underlying mechanism.

## 2 Materials and Methods

### 2.1 Animal Model Generation

All our experimental procedures were approved by the Ethics Committee of PLA General Hospital and performed in accordance with the Guide for the Care and Use of Laboratory Animals published by the U.S. National Institutes of Health (Publication No. 23, revised 1996). Mice were housed and cared for at PLA General Hospital Laboratory (Beijing, China). FGF23 floxed mice were generated based on CRISPR/Cas9 technology by Saiye Biotechnology Co., LTD (Suzhou, China). To obtain mice with cardiac myocyte‐specific FGF23 deletion (FGF23^f/f^MyHC^Cre/+^, FGF23‐CKO), FGF23 floxed C57BL/6J mice were crossed with transgenic MyHC‐Cre C57BL/6J mice. Transgenic MyHC‐Cre C57BL/6 J mice were used as controls. The study showed that the level of FGF23 protein from cardiomyocytes in the heart of healthy adult mice increased in an age‐dependent manner [20]. Furthermore, oestrogen can affect the expression and function of ion channels in arrhythmias, thereby affecting the calcium current of myocardial cells. Consequently, male mice aged 3–6 months with stable cardiac FGF23 protein levels were selected for the experiment.

### 2.2 Echocardiography

Echocardiography was performed using the Vevo 2100 Imaging System (FUJIFILM Visual Sonics Inc.) as previously described [21]. Anaesthesia was induced with 3% isoflurane and maintained with 1% isoflurane over a breathing mask. Echocardiographic views were acquired in the parasternal long axis in B‐ and M‐modes, and the short axis in M‐mode. All echocardiographic images were analysed using the Vevo Lab 3.2.0 software.

### 2.3 Intraesophageally Burst Pacing and Induction of AF

All experiments were performed as previously described [22]. Briefly, mice were anaesthetised with an intraperitoneal injection of 1% pentobarbital sodium, and their ECG recordings were instrumented with subcutaneous electrodes (Power Lab 16/35, ad Instruments, Castle Hill, NSW, Australia). Intraesophageal burst pacing was used to assess susceptibility to AF. The electrode was inserted through the oesophagus and placed at the site with the lowest threshold for atrial capture. Atrial pacing was performed at twice the diastolic threshold value using two poles on the pacing catheter. First, the pulse was paced for 10 s with a circle length of 100 ms, followed by burst stimulation for 10 s with a circle length of 30 ms until 10 consecutive burst stimulations or AF were induced. AF was defined as irregular, rapid atrial activation, with varying electrogram morphology lasting ≥ 10 s. All mice were allowed a 3 min of recovery in sinus rhythm between stimulations for respiratory and circulatory recovery. Given the difficulty in inducing AF in healthy mice using only tachypacing, an intraperitoneal injection of 1.5 mg/kg isoproterenol (Iso) (Sigma, USA) was performed to increase susceptibility to AF. The occurrence and duration of AF in each group were observed and recorded.

### 2.4 Adult Atrial Myocytes Isolation

As previously described, atrial myocytes were isolated using enzymatic digestion [23]. Briefly, the heart was quickly removed from the thorax. Subsequently, aortic cannulation was performed to flush the residual blood. The well‐perfused heart tissues were digested using trypsin(Gibco, USA) and collagenase(Worthington, USA). The Langendorff system facilitated the procedures described above. After digestion, the atrial myocytes were centrifuged and resuspended. Ca^2+^ was gradually reintroduced into the 10 mL cell suspension in a stepwise manner to avoid calcium paradox and calcium overload. Then, a total of 50 μL 100 mM CaCl_2_ (at every 5 min interval of 5, 10, 15 and 20 μL, respectively) was gradually added to the cell suspension. Atrial myocytes were transferred into a 1.8 mM Ca^2+^‐containing Tyrode's solution for measuring intracellular Ca^2+^ and membrane currents/potentials. The atrial myocytes were enzymatically isolated. After calcium reintroduction, there are about 70%–80% of myocytes that remain in the survival state. Only myocytes with a rod shape, clear striations, and stable contractions were selected for the experiment. All experimental procedures were limited to 6 h. The isolation solution used in this study contains (in mmol/L): 113 NaCl, 4.7 KCl, 0.6 KH_2_PO_4_, 0.6 Na_2_HPO_4_, 1.2 MgSO_4_, 12 NaHCO_3_, 10 KHCO_3_, 10 HEPES, 15 taurine, 5 glucoses and 10 2,3‐butanedione monoxime.

### 2.5 Isolation, Culture and Gene Transfer of NMAMs

Neonatal C57/BL6J mice (1–2 days old) were sacrificed by decapitation. Hearts were quickly excised, and the atria were dissected and transferred into ice‐cold Hank's balanced salt solution (HBSS) without Ca^2+^ and Mg^2+^ (Gibco, USA), then rapidly minced by three to five cuts with a fresh scalpel blade and placed in a 10 mL Penicillin vial containing 5 mL of enzymatic dissociation medium. The trypsin dissociation medium contained 0.08% trypsin (Gibco, USA) and 0.001 mg/mL collagenase II (Worthington, USA) dissolved in CMF HBSS. The collagenase dissociation medium contained 2 mg/mL collagenase (type 2; Worthington, USA) and 1 mg/mL BSA(Sigma, USA). The supernatant from each 5 min dissociation cycle was filtered through a 100‐μm cell strainer (Falcon, USA) in 5 mL cell culture media supplemented with 10% FBS (Gibco, USA)and 100 U/mL penicillin–streptomycin (Invitrogen, USA). After the fourth dissociation cycle at 37°C, the remaining tissue was triturated by gentle pipetting and strained, and the cell suspension was pelleted by centrifugation at 500 ***g*** for 10 min. The pellet was resuspended in 10 mL of culture media, and myocytes were enriched by 90 min of differential cell adhesion at 37°C in a 100‐mm culture dish. The supernatant was pelleted by centrifugation and then resuspended. Then 3 × 10^5^ NMAMs were seeded on 35‐mm cell culture plates coated with 0.5% gelatin (Sigma, USA)and cultured in DMEM containing 1 g/L glucose plus 15% FBS and 1% penicillin/streptomycin (Gibco, USA)for follow‐up experiments 48 h later.

FGF23 overexpression lentivirus vector was constructed by Saiye Biotechnology Co., LTD (Suzhou, China). FGF23 sequence was inserted into EGFP/T2A/Puro lentivirus vector and driven by EFS promoter. NMVMs were infected at a multiplicity of infection (MOI) of 50. Before transfection, the original medium was replaced with fresh and complete culture containing 10 μg/mL Polybrene (Sigma, USA)co‐transfection reagent, and the cells were pretreated for 30 min. After pretreatment, an appropriate amount of virus suspension was added according to the infection complex MOI determined in the previous pre‐experiment, and then put back into the cell incubator for further culture. 16 h after infection, fresh complete culture medium was replaced and continued to culture at 37°C. Two days after transfection, the cells were used for follow‐up experiments.

### 2.6 Patch Clamp Experiments

Patch‐clamp experiments were used to record action potentials and I_Ca,L_ in atrial myocytes. Membrane potential and membrane currents were recorded at the physiological temperature (35°C–37°C) using the patch‐clamp technique in whole‐cell recording mode. Temperature was controlled with a TC‐344C heater controller (Warner Instruments). A P70 horizontal puller (Sutter Instruments) was used to pull the borosilicate glass pipettes (World Precision Instruments) to a resistance of 2–4 MΩ. Membrane potentials were sampled at 10 kHz using a 1440A Digidata (Axon Instruments) controlled by pCLAMP programs (version 10.2). Cell capacitance, series resistance (compensation, 70%–80%), and junction potentials were compensated using the circuitry of the Axon Multiclamp 700B Amplifier (Molecular Devices, USA) and low‐pass filtered at 5 kHz. Protocols, data acquisition, storage, analysis, current fitting, and offline subtraction were performed using Clampfit 10.4 (Molecular Devices), and all curves were fitted with Origin (Microcal software).

Action potentials were recorded after applying a 2.5 ms/1 nA depolarization stimulation in the current‐clamp mode. Delayed after depolarizations were defined as the presence of spontaneous depolarization of the impulse after full repolarisation occurred. Micropipettes were filled with a solution containing (in mM) KCl 20, K aspartate 110, MgCl_2_ 1, Mg_2_ATP 5, HEPES 10, EGTA 0.5, NaGTP 0.1 and Na_2_ phosphocreatine 5, titrated to a pH of 7.2 with KOH. Cells were bathed in a solution that contained NaCl 140 mM, CaCl_2_ 1 mM, MgCl_2_ 1 mM, HEPES 10 mM, KCl 4 mM and glucose 5 mM. Its pH value was 7.36 and adjusted with CsOH.

I_Ca,L_ was recorded in the voltage‐clamp mode from a holding potential of 80 mV. Pulse‐60 mV was depolarized to +50 mV with a 10 mV voltage step, and the depolarization pulse was set to 200 ms. These recordings were used to plot the current voltage relationship (*I‐V* curve), steady‐state activation (SSA), or steady‐state inactivation (SSI) curves. To record the I_Ca,L_, the pipette solution contained CsCl 120 mM, CaCl_2_ 1 mM, MgCl_2_ 5 mM, EGTA 11 mM, HEPES 10 mM, and Na_2_ATP 5 mM, and was adjusted to pH 7.2 with CsOH. The external solution contained NaCl 140 mM, CaCl_2_ 2 mM, MgCl_2_ 1 mM, KCl 4 mM, HEPES 10 mM and glucose 10 mM and was adjusted to pH 7.4 with CsOH. Tetrodotoxin (5 μM) was added to block sodium current when recording calcium currents. Current amplitude data of each cell were normalised to its cell capacitance (current density, pA/pF), and the *I‐V* curve was plotted. Voltage‐dependent activation and steady‐state inactivation profiles were fitted to a Boltzmann equation a = 1/{1 + exp. [−(*V*m‐*V*1/2)/k]}, where a is the normalised conductance, *V*m is the test potential, *V*½ is the potential at which current is half activated/inactivated, and *k* is the slope factor. Electrophysiological data were analysed through Clampfit 10.4 (Axon Instruments) and Origin (Microcal software).

### 2.7 Cardiomyocyte Ca^2+^ Imaging

Isolated atrial myocytes were incubated with 2 μmol/L Fluo‐4 AM (Invitrogen, USA) for 20 min and then equilibrated in fresh Tyrode's solution with 1.8 mM Ca^2+^ containing 250 μmol/L probenecid (Sigma, USA) for 20 min to allow dye de‐esterification in laminin‐coated dishes. The Myocyte Calcium & Contractility Recording System (IonOptix, Westwood, MA, USA) was used to record transient changes in Ca^2+^ concentrations. Atrial myocytes were pre‐conditioned with field‐stimulating at 1 Hz to achieve a steady state of at least 20 beats, and Ca^2+^ sparks were acquired over a 10‐s rest period. For determination of SR Ca^2+^ load, rapid delivery of 10 mM caffeine was used. Diastolic events (sparks) were obtained using confocal microscopy (SP5, Leica Microsystems, German). Only recordings that showed no spontaneous Ca^2+^ waves were included in the analysis.

### 2.8 Western Blotting

Western blotting was used to investigate the expression of calcium transport‐related proteins. The left atrial tissue was collected from liquid nitrogen and lysed in RIPA buffer (Solarbio, China). Protein content was quantified using a BCA reagent kit(Solarbio, China). Protein samples from each group were separated by 4%–12% SDS‐PAGE and then transferred to PVDF membranes (Millipore, USA). The membranes were blocked with 5% non‐fat milk (Sigma, USA) for 1 h at room temperature and then incubated with the specified primary antibody overnight at 4°C (anti‐RyR2, 1:1000 dilution, Abcam, UK; anti‐Cav1.2, 1:1000 dilution, Abcam, UK; anti‐NCX1.1, 1:1000 dilution, Abcam, UK; anti‐SERCA2a, Abcam, UK, 1:1000 dilution; anti‐FGF23, 1:2000 dilution, R&D, USA; anti‐GAPDH, 1:5000 dilution, Abcam, UK). After being washed three times with TBST, the membranes were incubated with the secondary antibody (1:20000 dilution, ZSBIO, China) at room temperature for 1 h. Particular signals were revealed by the chemiluminescence detection reagent Western lightning plus‐ECL. GAPDH (1:5000 dilution, Proteintech, USA) was used to normalise the protein sample loading. Finally, ImageJ software was used to analyse the gel images.

### 2.9 RNA Isolation and qRT‐PCR Analysis

A Cell Total RNA Isolation Kit (FOREGENE, China) was used to prepare total RNA from NMAMs. cDNA was generated using a reverse‐transcription system (Thermo Fisher, USA). The obtained cDNA was amplified using a SYBR Premix Kit (Thermo Fisher, USA) on a BIO‐RAD CFX96 Real‐Time PCR System (Bio‐Rad Laboratories, USA). Relative gene expression values were calculated with the $2^{-{\mathrm{\Delta \Delta C}}_{t}}$ method using GAPDH as a housekeeping gene.

### 2.10 Statistical Analysis

Continuous variables were presented as mean ± standard error (SE). One‐way ANOVA with Bonferroni post hoc analysis or student's *t*‐test was used for comparison between groups. Fisher's exact test was used to compare the incidences of atrial tachyarrhythmia and oscillation in membrane potentials (DADs) between the groups. SPSS software (version 20.0) was used for data analysis. Statistical significance was set at *p* < 0.05.

## 3 Results

### 3.1 Mice With Cardiac Myocyte‐Specific Knockout of FGF23 Reveal Normal Cardiac Function

**FIGURE 1:**
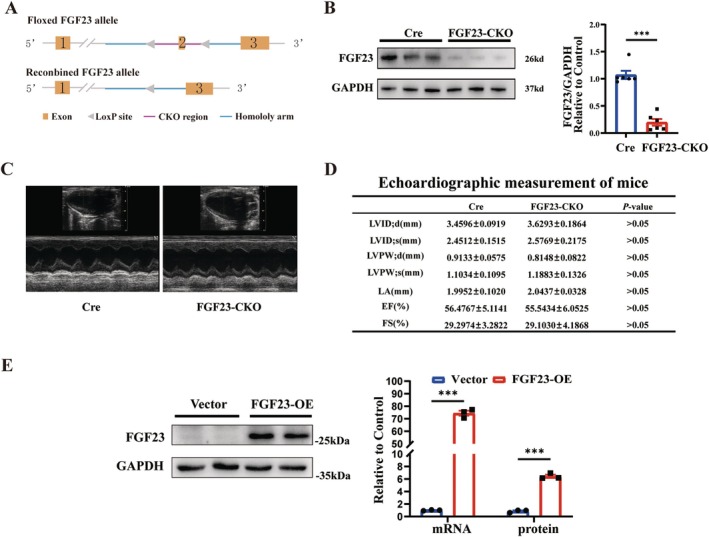
Characterisation of the cardiac myocyte‐specific FGF23 knockout mice. (A) Schematic illustration of Cre‐LoxP strategy to generate mice with cardiac myocyte specific FGF23 knockout showing murine FGF23 allele with exons 1–3 and location of LoxP sites (triangles) surrounding exon 2. Top: Floxed FGF23 allele, bottom: FGF23 allele after Cre‐mediated recombination. (B) Representative immunoblot and respective quantification show significantly reduced FGF23 protein levels in total heart lysates of FGF23‐CKO compared to control mice. GAPDH serves as loading control. (C) Representative parasternal long‐axis M‐mode echocardiography images of FGF23‐CKO and Cre mice. (D) Quantification of heart function using echocardiography, respectively, in FGF23‐CKO and Cre mice. (E) FGF23 mRNA and protein expression were increased markedly in overexpressed neonatal mouse atrial myocytes models.

Whole‐gene FGF23 knockout mice survived no more than 13 weeks of age [24]. A cardiac myocyte‐specific FGF23 knockout mouse model was established. Cardiac‐derived FGF23 is in more detail in the development of AF (Figure 1A). The two LoxP sites were respectively inserted upstream and downstream of exon 2, which was deleted after recombination enzyme activation. Compared with Cre mice, western blotting showed lower FGF23 levels in cardiomyocytes of conditioned knockout mice (Figure 1B). The parameters of cardiac structure were measured by echocardiography (Figure 1C); there was no difference in cardiac function after FGF23 conditional knockout (Figure 1D).

Following infection of C57Bl/6J mice atrial myocytes with FGF23‐overexpressing lentiviral vector (FGF23‐OE) and control vector, qRT‐PCR and Western Blot analysis showed that upon overexpression of FGF23, the expression of FGF23 mRNA and FGF23 protein in rat atrial myocytes was significantly increased (Figure 1E).

**FIGURE 2:**
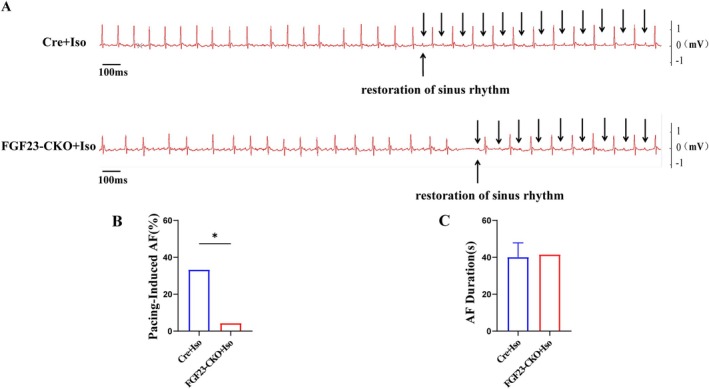
Representative simultaneous recordings of surface ECG in FGF23‐CKO mice and control mice following programmed stimulation. (A) Representative recordings of surface ECG in FGF23‐CKO mice and control mice induced atrial fibrillation following burst stimulation. Black arrows showed sinus rhythm. (B) Bar graph summarising the incidence of inducible AF in FGF23‐CKO mice after intraperitoneal injection of isoproterenol. Data were analysed using Fisher's exact test (*n* = 23 mice for FGF23‐CKO and *n* = 15 mice for Cre, *p < 0.05 vs. Cre + Iso). (D) No difference was found in duration of AF between two groups (*n* = 23 mice for FGF23‐CKO and *n* = 15 mice for Cre).

Decreases the susceptibility to AF in FGF23‐CKO mice, as shown in Figure 2A. AF was observed after rapid transesophageal atrial pacing and could spontaneously convert to a normal sinus rhythm shortly thereafter. After acute intraperitoneal injection of Iso (1.5 mg/kg) for 15 min, the incidence of AF in the FGF23‐CKO group (4.34%, *n* = 1/23) was significantly decreased compared with the Cre group (33.3%, *n* = 5/15) (*p* < 0.05) (Figure 2B). Figure 2C is a local magnification diagram of the recovery of sinus rhythm from AF rhythm in Figure 2A. No difference was found in the duration of AF between the two groups (FGF23‐CKO 41.45 ± 0 s vs. Cre 40.08 ± 7.73 s, *p* > 0.05) (Figure 2C).

### 3.2 Increased TA in FGF23 Overexpression of NMAMs

**FIGURE 3:**
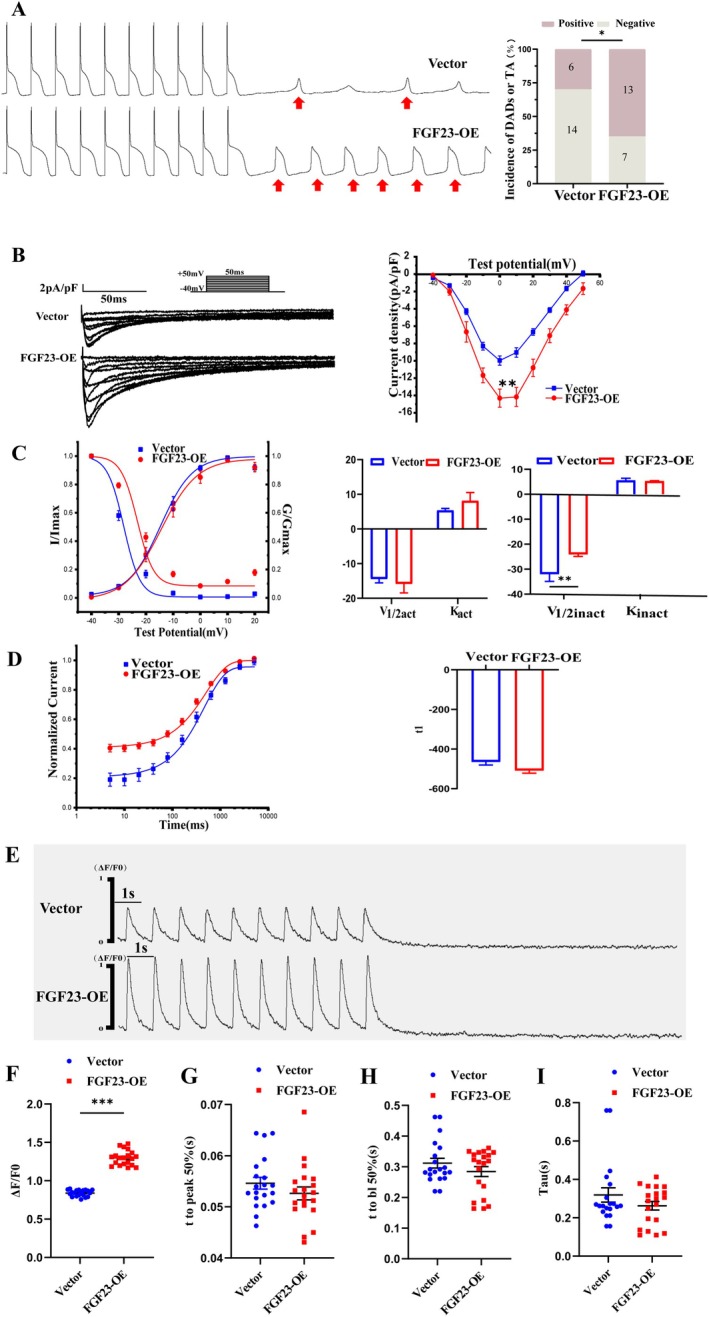
Characteristics of cellular electrophysiology and Ca^2+^ transients in FGF23 overexpression of NMAMs. (A) Raw diagram of Action potentials recorded in two groups. FGF23 overexpression of NMAMs exhibited a higher incidence of triggering activity (Red arrow) than the control. (*n* = 20/6 ~ 10 cells/neonatal mice, *p < 0.05 vs. Vector，by Student's *t*‐test). (B) Raw diagram of L‐type calcium current in two groups (left). Overexpression of FGF23 increased the peak density of I_Ca,L_ in neonatal mouse atrial myocytes (right). (*n* = 10/6 ~ 10 cells/neonatal mice, **p < 0.01 vs. Vector，by Student's *t*‐test) (C) SSA and SSI for I_Ca,L_ in neonatal mouse atrial myocytes of overexpressing FGF23. The SSI curve of. FGF23‐overexpression group shifts significantly to the right. (*n* = 15/6 ~ 10 cells/neonatal mice, **p < 0.01 vs. Vector，by Student's *t*‐test) (D) The recovery kinetics process after calci‐electric loss accelerated when FGF23 was overexpressed. (*n* = 10/6 ~ 10 cells/neonatal mice, *p* > 0.05) (E) The cardiomyocytes of FGF23‐Overexpression and Vector groups were stimulated at 1 Hz and recorded for 10 s after the stimulation. (F) The calcium transient amplitude in the FGF23‐Overexpression group was significantly increased. (G–I) There was no significant difference in t to peak 50% (bl%) and calcium release decay time (peak time) between FGF23‐Overexpression and Vector groups. (*n* = 20/6 ~ 10 cells/neonatal mice, ***p < 0.001 vs. Vector，by Student's t‐test).

A FGF23‐overexpression model of neonatal cardiomyocytes was established. Atrial myocytes in each group were electrically stimulated under the current clamp mode, and a higher incidence of triggering activity was observed in FGF23 overexpression of NMAMs than in the vector, which may result in the occurrence of AF (FGF23‐OE 65% (13/20) vs. Vector 30% (6/20), *n* = 20/6 ~ 10 cell/neonatal mice, *p* < 0.05) (Figure 3A).

### 3.3 Increased I_Ca,L_ in FGF23 Overexpression of NMAMs

After overexpression of FGF23, I_Ca,L_ were significantly increased in neonatal atrial myocytes in mice (FGF23‐OE −14.17 ± 1.09 pA/pF vs. Vector 9.96 ± 0.53 pA/pF, *n* = 10, *p* < 0.05) (Figure 3B). The gating mechanism characteristics of I_Ca,L_ showed that the overexpression of FGF23 had no effect on the activation curve of calcium currents. SSI half‐inactivation voltage of calcium current shifted to the right, with a statistically significant difference (*p* < 0.05) (Figure 3C). The recovery kinetics process after calci‐electric loss was accelerated when FGF23 was overexpressed in studies on the recovery kinetics after the inactivation of I_Ca,L_ (Figure 3D).

### 3.4 Increased Ca^2+^ Transients in FGF23 Overexpression of NMAMs

Calcium release and uptake in two groups of NMAMs were recorded (Figure 3E). The amplitude of calcium release was significantly increased after overexpression of FGF23 in neonatal atrial myocytes (FGF23‐OE 1.29 ± 0.02 vs. Vector 0.83 ± 0.01, *n* = 20, *p* < 0.05) (Figure 3F). Calcium release peaked half the time; calcium elimination half time and calcium elimination time constant did not differ significantly (Figure 3G–I).

### 3.5 Decreased DADs in Atrial Myocytes of FGF23‐CKO Mice

**FIGURE 4:**
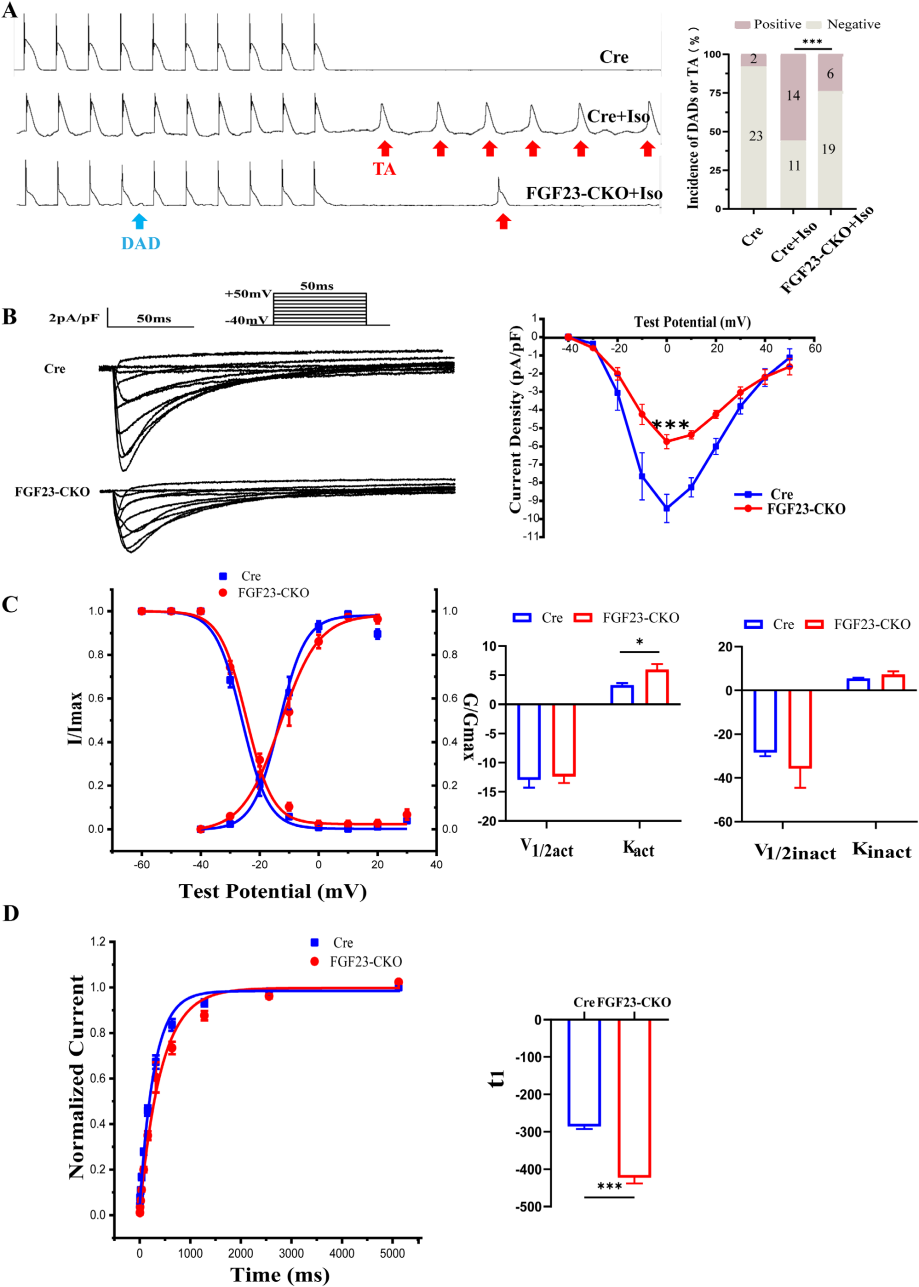
Cellular electrophysiological characteristics of adult atrial myocytes in Cre and FGF23‐CKO mice. (A) FGF23‐CKO had a lower occurrence of delayed afterdepolarizations (blue arrow) or Trigger activities (Red arrow) than control after Iso inducing. (*n* = 25/3 cells/mice, ***p < 0.001 vs. Cre，by Student's *t*‐test). (B) I_Ca,L_ current traces in 10 mV increments from a holding potential of −40 mV to +50 mV in the Cre and FGF23‐CKO atrial myocytes(left). I‐V curve for I_Ca,L_. At 0 mV, the peak density of I_Ca,L_ of FGF23‐CKO decreased from −9.42 ± 0.78pA/pF to −5.75 ± 0.39 pA/pF (right). (*n* = 10/3 cells/mice, ***p < 0.001 vs. Cre，by Student's *t*‐test) (C) SSA and SSI for I_Ca,L_ in the FGF23‐CKO and Cre group. The slope factor K_act_ of the FGF23‐CKO group were significantly increased. (*n* = 10/3 cells/mice, ***p < 0.001 vs. Cre，by Student's *t*‐test) (D) The recovery curve after inactivation between the two groups, the t1 of the FGF23‐CKO were significantly higher than that of the Cre group. (*n* = 10/3 cells/mice, ***p < 0.001 vs. Cre，by Student's *t*‐test)

To verify whether FGF23‐CKO mice have a similar phenotype to neonatal cardiomyocytes, the atrial myocytes were isolated from adult mice to observe the above‐mentioned indexes. The incidence of DAD/TA in the FGF23‐CKO group was significantly decreased after Iso treatment of atrial myocytes (FGF23‐CKO + Iso 24% (6/25) vs. Cre + Iso 56% (14/25), *p* < 0.05) (Figure 4A).

### 3.6 Decreased I_Ca,L_ in Atrial Myocytes of FGF23‐CKO Mice

I_Ca,L_ was recorded in two groups of atrial myocytes in voltage clamp mode (Figure 4B). The current density was obtained by current amplitude/film capacitance (pA/pF), and the *I‐V* curve of I_Ca,L_ was obtained by plotting the current density against the stimulation voltage. As shown in Figure 4B (right), the *I‐V* curve in FGF23‐CKO group is significantly upward compared with the control group. The I_Ca,L_ current densities decrease significantly between −30 mV and + 30 mV. The shape of *I‐V* curves in the two groups does not change, showing a typical ‘inverted bell shape’. When the clamping voltage is 0 mV in both groups, the current density reaches the maximum, and the I_Ca,L_‐peak current density of FGF23‐CKO mice in the control group decreases from −9.42 ± 0.78 pA/pF to −5.75 ± 0.39 pA/pF (*n* = 10, *p* < 0.05).

The I_Ca,L_ gating mechanism showed an increased slope *k* factor of the steady‐state activation curve of FGF23‐CKO mice (FGF23‐CKO 3.30 ± 0.34 vs. Cre 5.97 ± 0.91, *n* = 15, *p* < 0.05) (Figure 4C (middle)). There was no significant difference in the half‐inactivation voltage and slope factor of the steady‐state inactivation curve (Figure 4C (right)). The recovery rate of I_Ca,L_ in FGF23‐CKO mice was decelerated after inactivation, and the recovery time constant increased in studies on the recovery kinetics of I_Ca,L_ (FGF23‐CKO −169.21 ± 6.26 ms vs. Cre −114.33 ± 2.50 ms, *n* = 10, *p* < 0.05) (Figure 4D).

### 3.7 Reduced Systolic Ca^2+^ Transients, Diastolic Spontaneous Ca^2+^ Leak in Atrial Myocytes of FGF23‐CKO Mice

**FIGURE 5:**
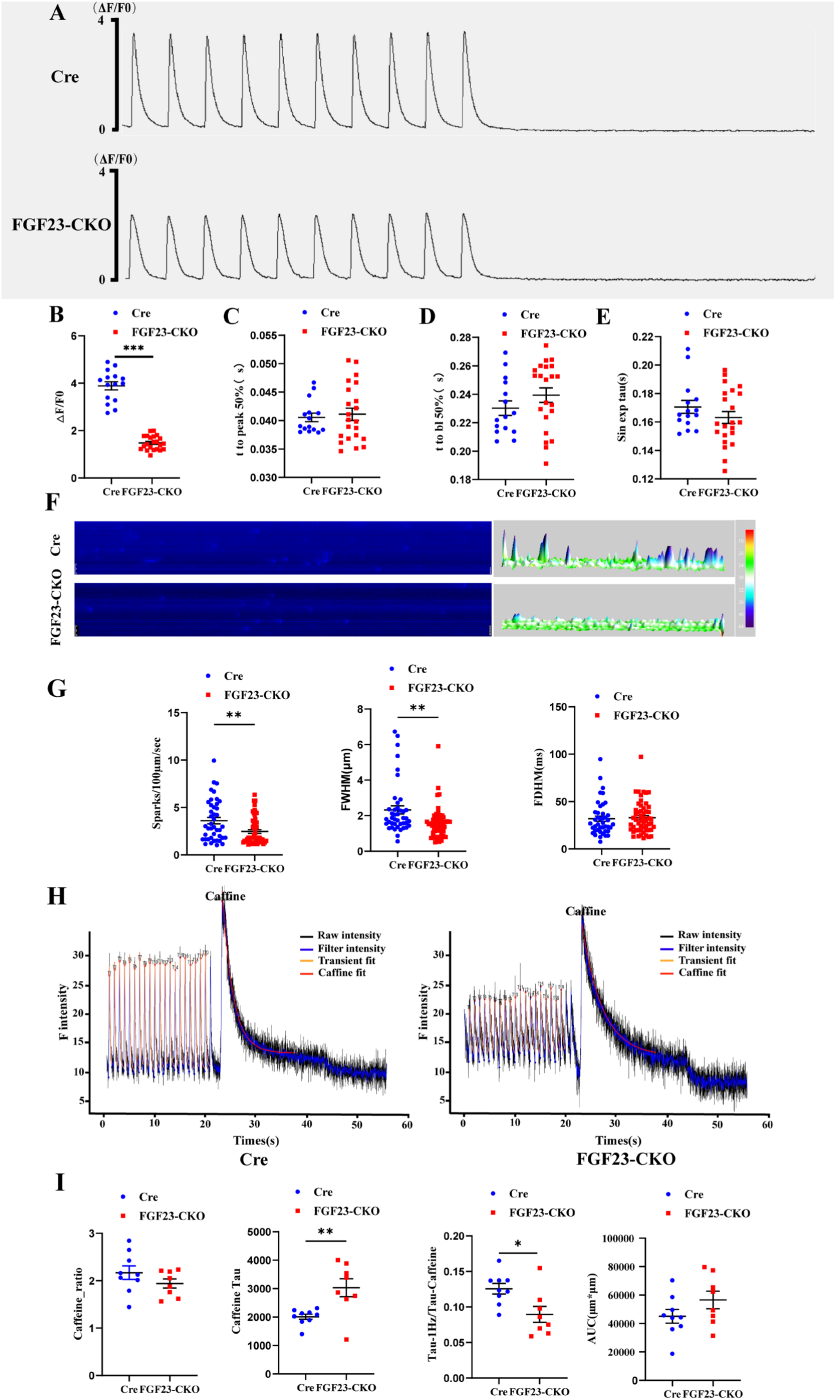
Effects of cardiac FGF23 on intracellular calcium in adult atrial cells. (A) The cardiomyocytes of the adult two groups were stimulated at 1 Hz and recorded for 10 s after the stimulation. (B) The calcium transient amplitude in the FGF23‐CKO group was significantly decreased. (*n* = 22/3 cells/mice, ***p < 0.001 vs. Cre *n* = 15/3 cells/mice，by Student's *t*‐test) (C‐E) There was no significant difference in t to peak 50% (bl%) and calcium release decay time (peak time) between the two groups. (F) Representative line scan images of Ca^2+^ sparks recorded in a cardiomyocyte adult intact mouse atrial myocyte and 3D imaging of Ca^2+^ sparks. (G) Bar graphs comparing the Ca^2+^ spark frequency (CaSpF), FWHM and FDHM between the Cre and FGF23‐CKO groups. The FGF23‐CKO group showed significant decreased values of Ca^2+^ spark frequency and FWHM. FWHM: Full width half maximum, FDHM: Full duration half maximum. *n* = 54/3 cells/mice for FGF23‐CKOand *n* = 42/3 cells/mice for Cre, Value = Mean ± SEM, t‐test were performed, ** *p* < 0.01 compared with the Cre group. (H) Raw diagram of SR calcium release induced by Caffeine of two groups. (I) Bar graphs comparing the Caffeine ratio, Tau‐caffeine and Tau‐1 Hz/Tau‐caffeine between the Cre and FGF23‐CKO groups. The FGF23‐CKO group showed significant increased values of Tau‐caffeine and decreased values of Tau‐1 Hz/Tau‐caffeine than the Cre group. *n* = 9/3 cells/mice for FGF23‐CKO and *n* = 8/3 cells/mice for Cre Value = Mean ± SEM, t‐test were performed, **p < 0.01, *p < 0.05 compared with the Cre group.

The calcium release amplitude of FGF23‐CKO mice was decreased compared with that of Cre mice (FGF23‐CKO: 1.48 ± 0.06 *n* = 15 vs. Cre 2.88 ± 0.14, *n* = 22, *p* < 0.05) (Figure 5A,B) There was no significant difference in half of the time for calcium release to reach its peak, half of the time for calcium elimination, and the elimination time constant (Figure 5C–E).

In addition, the effect of cardiac FGF23 on diastolic calcium disposal was observed. The calcium spark frequency in the FGF23‐CKO group was reduced (Figure 5F,G (left)). FDHM remained unchanged (Figure 5G (right)) and FWHM was decreased in the study on Calcium spark kinetics (Figure 5G (middle)).

The effect of cardiac FGF23 on SR calcium content was studied. After field‐stimulating atrial cardiomyocytes to reach the steady state, caffeine was applied to deplete the SR of calcium (Figure 5H). There was no significant difference in the amplitude of caffeine‐evoked calcium transients, an estimation of SR calcium load between the two groups (Figure 5I). The time constant of calcium elimination was increased when Caffeine was continuously injected in the FGF23‐CKO group (Figure 5I (middle)).

### 3.8 Changes in Expression of Ca^2+^ Handling Proteins in Adult Atrial Myocytes of FGF23‐CKO

**FIGURE 6:**
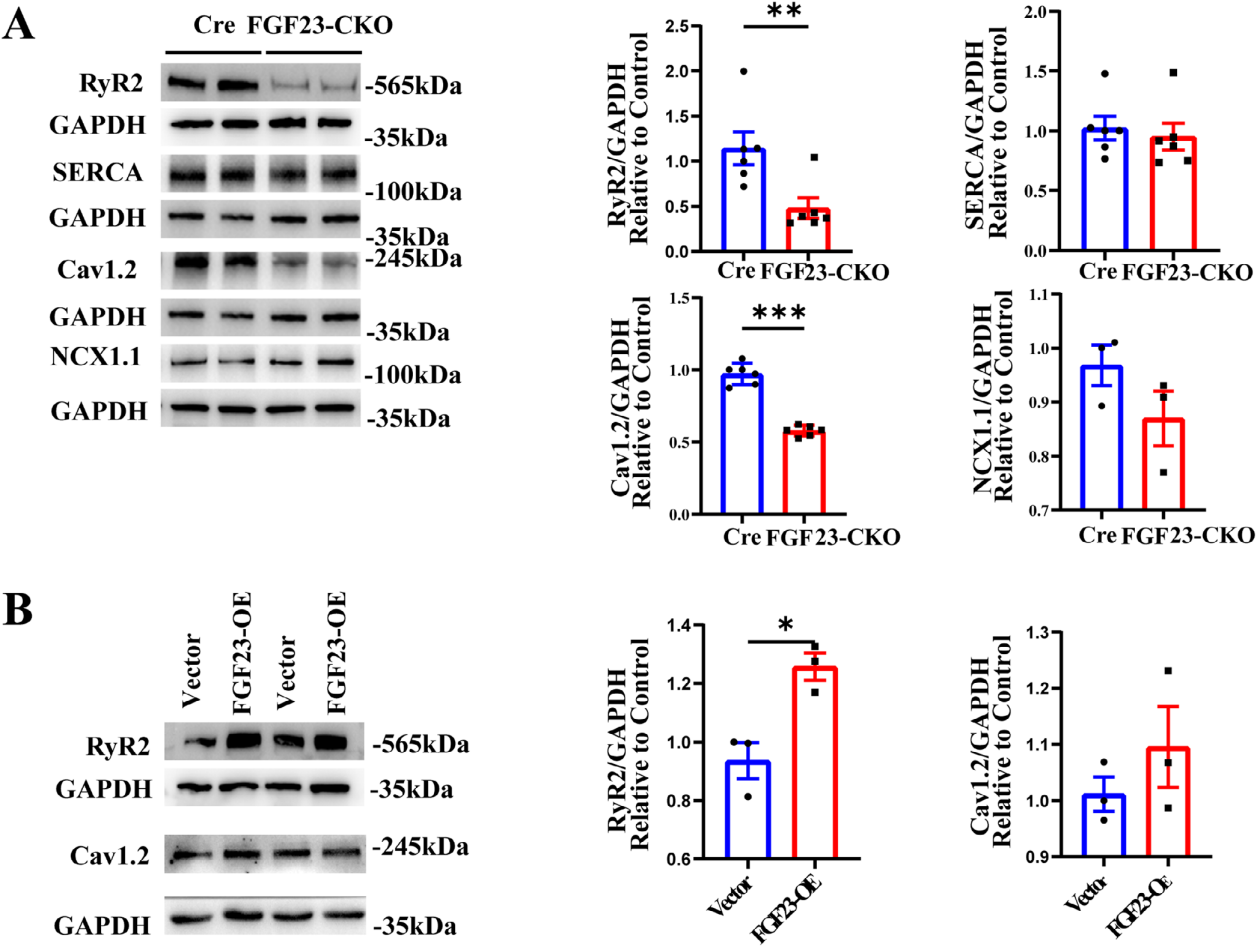
Calcium channel and transporter protein expressing in adult and neonatal mice atrial myocytes. (A) Representative western blots and quantification of Ryanodine Receptor 2 (RyR2), SRECA2a, Cav1.2 and NCX1.1 expression in the atrial tissues of mice in the FGF23‐CKO group and Control group with GAPDH as a loading control (*n* = 6 mice per group). Values are presented as mean ± SE. **p < 0.01, ***p < 0.01 vs. Cre group. (B) Representative western blots and quantification of Ryanodine Receptor 2 (RyR2), Cav1.2 expression in neonatal mice atrial myocytes in the FGF23‐OE group and Vector group with GAPDH as a loading control (*n* = 3 per group). Values are presented as mean ± SE. *p < 0.05 vs. Vector group.

The effects of FGF23‐CKO on calcium handling proteins were detected in mouse atrial muscle tissue of two groups. FGF23‐CKO and Cre mice shared similar expression of SERCA2a protein, which is responsible for SR calcium uptake (Figure 6A). However, compared with Cre mice, Lower expression of RyR2 protein responsible for calcium release in the SR was presented in FGF23‐CKO mice (Figure 6A). Additionally, a significant 80% decrease in the expression of Cav1.2, a cell membrane protein responsible for calcium handling, was observed in the FGF23‐CKO group, while there was no significant change in the expression of NCX1.1 (Figure 6A). Consistently, the expression of RyR2 protein was up‐regulated, but the protein level of Cav1.2 remained unchanged in the FGF23‐overexpressed NMAMs (Figure 6B).

## 4 Discussion

Previous studies have demonstrated an extremely low rate of AF induced by burst stimulation in healthy young mice, making AF induction challenging [25]. In this study, Iso was used to increase the susceptibility of mice to AF. The incidence of AF was increased to 33.3% in the control Cre group, while that was only 4.34% in the FGF23‐CKO group, suggesting that FGF23‐CKO mice were less susceptible to AF under the condition of acute Iso‐induced β adrenergic stress.

Electrophysiological recordings from two groups of mice revealed that the incidence of Iso‐induced DAD or TA was significantly reduced in FGF23‐CKO mice as compared with the Cre group. Isoproterenol, by binding to cardiomyocyte β receptors, activates calcium channels through the intracellular AC‐cAMP‐PKA signalling pathway, thus increasing calcium inflow and enhancing the autotrophic conductive contractility of cardiomyocytes. Our previous studies have demonstrated that Iso can increase the influx of calcium ions in L‐type calcium channels and enhance the sensitivity of RyR2 channels in SR, which further promotes calcium ion leakage during diastole. As a result, intracellular calcium ions are increased to activate the activity of sodium‐calcium exchange bodies in the cell membrane, thereby generating instantaneous inward Iti current and increasing the occurrence of DAD [26]. Relevant studies have shown that DAD‐mediated TA contributes to the occurrence of AF [9], which explains that a decrease in the incidence of AF in the FGF23‐CKO group is due to the reduction of the incidence of ISO‐induced DAD. Conversely, opposite results were found after overexpression of FGF23 in neonatal atrial myocytes in the present study. The incidence of TA was increased when FGF23 was overexpressed in atrial myocytes. Unlike adult cardiomyocytes, neonatal mouse cardiomyocytes undergo a more rapid dedifferentiation‐redifferentiation cycle, typically resulting in spontaneous beating of the cells 20 h after plating, while adult cardiomyocytes typically require pacing to induce contraction. Therefore, newborn mouse cardiomyocytes do not need to be induced by drugs to generate triggering activity.

DAD results from the disturbance of intracellular calcium circulation. Physiologically, when cardiomyocytes depolarise, a small amount of extracellular calcium ions rapidly flows into the cell through the L‐type calcium channel in the cell membrane. Calcium ions bind to the RyR2 receptor in the SR to open the calcium channel, resulting in the release of a large amount of calcium ions from the SR into the cytoplasm, which causes the contraction of cardiomyocytes. This process is called calcium‐induced calcium release (CICR) [27, 28]. During myocardial diastole, excessive intracellular calcium ions are removed through two main mechanisms: reuptake into the SR through the sarcoplasmic reticulum calcium ATPase pump (SERCA), or excretion of intracellular calcium ions into the extracellular through the membrane sodium‐calcium exchange (NCX) [29]. Any problem in any of these links can lead to the disturbance of intracellular calcium circulation.

Consequently, further investigation into intracellular calcium disposal revealed that the peak calcium current and I_Ca,L_ inflow were significantly reduced in the FGF23‐CKO group. The gating mechanism of I_Ca,L_ in the FGF23‐CKO group exhibited an increased slope factor of the activation curve, indicating that I_Ca,L_ activation was slowed down, and the number of active channels activated was decreased at the same voltage. Additionally, it was observed that the recovery time after the inactivation of L‐type calcium channels was prolonged, which is the gating mechanism for the reduction of I_Ca,L_. Immunoblotting showed that the protein expression of Cav1.2 in the atrial muscle tissue of the FGF23‐CKO group was nearly 40% lower than that of the Cre group. Coupled with the above reasons, it mediated a reduction in I_Ca,L_, thereby reducing the inflow of intracellular calcium ions and maintaining a low intracellular calcium environment. After overexpression of FGF23, the peak current density of I_Ca,L_ was significantly increased. The gating mechanism of I_Ca,L_ showed that the inactivation curve moved significantly towards depolarization, and the inactivation of I_Ca,L_ was slowed down, suggesting an increase in the number of effective channels of I_Ca,L_ under the same voltage. Despite no difference in the recovery time constant, the recovery curve after overexpression of FGF23 was significantly shifted to the left after inactivation. These results indicate that the inflow of calcium ions into L‐type calcium channels, as well as the intracellular calcium ion level, can be increased by cardiac FGF23. However, there was no significant difference observed in the protein level of Cav1.2 in vitro, which was inconsistent with the results in vivo. Furthermore, The results demonstrated that there were obvious effects on the gating mechanism of I_Ca,L_ in both vivo and vitro groups, suggesting that cardiac FGF23 mainly increases intracellular calcium influx by regulating the gating mechanism of L‐type calcium channels.

In 2013, Touchberry et al. [17] found that intracellular calcium ion levels could be increased in cardiomyocytes treated with FGF23 in vitro and further found that the increase of intracellular calcium ions could be blocked by verapamil, a calcium channel blocker. This strongly suggests that the increase in intracellular calcium ions may be due to increased inflow of I_Ca,L_. In 2014, Kao et al. [30] also found that FGF23 could increase I_Ca,L_ in HL‐1 atrial cells, but they did not further explore changes in mechanisms underlying the changes in I_Ca,L_. Graves et al. found that FGF23 perfusion into the heart can cause the increase of intracellular calcium in the ventricular myocytes, which indicates that FGF23 can open the calcium channel of the cell membrane to promote the inflow of calcium ions and thus cause the increase of intracellular calcium, which produces similar results to this experimental study. However, the objects of their study were mainly ventricular muscle strips and isolated hearts, not single cardiomyocytes. The results may also be influenced by the effects of FGF23 on other cells and cell–cell interactions. In addition, they found that FGF23 can induce a prolonged QTc interval, suggesting that FGF23 may increase the risk of arrhythmia by affecting the repolarization process of cardiomyocytes. The continuous inflow of L‐type calcium current can prolong the action potential plateau, which may be one of the reasons for the prolongation of QTc interval caused by FGF23. However, the repolarization of cardiomyocytes is also related to other ions such as potassium ion, which may be the result of the combined action of multiple ion currents. The action potential plateau of ventricular muscle is longer than that of atrial muscle cells, and FGF23 may be more likely to cause repolarization‐related Ventricular arrhythmia. However, in 2019, findings from Navarro‐Garcia et al. [31] were inconsistent with ours. They demonstrated that FGF23 in vitro treatment of cardiomyocytes reduced I_Ca,L_. This discrepancy may be attributed to the inconsistent concentration and treatment time of FGF23 used for treating cardiomyocytes. Variations in I_Ca,L_ may be dose‐dependent and time‐dependent.

Simultaneously, detection of intracellular calcium ion release showed that the transient amplitude of calcium in the FGF23‐CKO group was decreased under 1 Hz stimulation, indicating a reduction in calcium‐induced instantaneous calcium ion flow triggered by calcium ion inflow, but the recovery function of calcium ions in the SR was not affected. The expression of RyR2 protein in atrial muscle tissue of the FGF23‐CKO group was observed to be lower than that of the Cre group. A reduction of calcium ions was released from the SR during the systolic period by decreasing the expression of RyR2 in the FGF23‐CKO group. The opposite results were observed after the overexpression of FGF23 in neonatal mouse atrial myocytes, with an increase in the amplitude of calcium transient and the expression of RyR2 protein. RyR2 typically remains closed during diastole, but a small portion of RyR2 channels will spontaneously open during diastole. In this study, the frequency of calcium spark during the diastolic period of FGF23‐CKO mice was reduced. Calcium spark dynamics showed that FDHM remained unchanged and FWHM was decreased, indicating that the diffusion range of calcium spark was mainly affected in the diastolic period rather than the duration in FGF23‐CKO mice. Decreased RyR2 expression results in reduced distribution density of RyR2 in the SR, thereby decreasing the spreading efficiency of RyR2 clusters. Numerous studies have shown that in addition to the increased sensitivity and instability of RyR2 leading to increased calcium ion leakage during diastole, non‐calcium spark mediated diastolic calcium leakage also plays a certain role. The underlying mechanism lies in that a single RyR2 spontaneously opens in the RyR2‐releasing cluster, while other channels in the cluster remain inactive [32], isolated non‐clustered RyR2 is activated [33]. A decrease in the expression of RyR2 can also reduce non‐calcium spark‐mediated RyR2 calcium leakage during the diastolic period, thereby reducing the occurrence of AF. Increased amplitude of calcium transients and increased load of calcium ions in SR after FGF23 treatment of HL‐1 cells were also shown in previous studies, which is consistent with the results of this study [34]. In the caffeine‐induced release of calcium pool, there was no difference in the SR calcium pool capacity between the two groups. The magnitude of calcium transients is related to the SR calcium load [33], Extracellular calcium inflow [35] and SERCA activity can affect SR calcium load [36]. An increase in SR load can increase the frequency of calcium sparks during diastole, thereby enhancing the activity of NCX during diastole and increasing the occurrence of DAD, which promotes AF. We observed that the calcium elimination time constant increased and calcium recovery slowed down in the FGF23‐CKO group after caffeine‐induced calcium release. During the 1 Hz stimulation phase, the decrease in calcium transients is attributed to SERCA in the SR and NCX in the cell membrane, and the decay time constant of calcium transients (Tau‐1 Hz) reflects the combined activity of SERCA and NCX. SERCA failed to build up SR calcium reserves during the caffeine infusion phase. Thus, the decrease in caffeine‐induced calcium pulses (Tau‐caffeine) was primarily attributed to NCX, which indicated a weakening of NCX function in the FGF23‐CKO group. However, this study did not find any difference in the level of NCX protein. It is evident that the quantity of NCX remained unchanged, but the function of NCX was weakened, thereby reducing the instantaneous inward Iti current and the occurrence of DAD in FGF23‐CKO mice. However, no significant differences were observed in sarcoplasmic reticulum calcium load between the two groups of mice, which may be attributed to the fact that the experiment was conducted under physiological conditions, and the impact of cardiac FGF23 on calcium load under pathological conditions remains unexplored.

Despite numerous studies conducted in various laboratories assessing the intracellular calcium ion environment, it has been consistently demonstrated that FGF23 can increase the intracellular calcium ion concentration. However, there is no comprehensive report in relevant literature targeting the specific source of calcium ions at the current stage, and the impact of FGF23 on I_Ca,L_ and RyR2 remains controversial. Kao et al. [30] found that 25 ng/mL of FGF23 could increase I_Ca,L_ in HL‐1 atrial cells and heighten the amplitude of calcium release, which further increases the calcium ion load of SR. However, Navarro‐Garcia et al. [31] used 100 ng/mL of FGF23 to irrigate adult Wister rat cardiomyocytes, and decreases in I_Ca,L_ of cardiomyocytes, the amplitude of calcium release, and the SR calcium load were observed. It was also found that the calcium spark frequency of cardiomyocytes was increased when incubated with FGF23. The results were completely inconsistent, and a reasonable explanation was that FGF23 embraced different acute and long‐term effects on cardiomyocytes. FGF23, like other well‐defined stress hormones such as norepinephrine, epinephrine, and angiotensin II, can increase I_Ca,L_ inflow and excitocontractile coupling to improve myocardial contractile force. However, long‐term exposure to FGF23 may lead to the disturbance of calcium circulation, which in turn activates the transcriptional remodelling mechanism, resulting in long‐term functional impairment and ultimately leading to heart hypertrophy [17]. The continuous inflow of I_Ca,L_ can increase the SR calcium load. When the sarcoplasmic reticulum load exceeds the threshold, the diastolic calcium ion leakage will also be increased. Meanwhile, pathways such as CAMKII [37] and pathways that promote the formation of intracellular ROS [38] can be activated by FGF23 to increase the opening probability of RyR2. The increase in diastolic calcium leakage aggravates intracellular calcium overload, leading to a gradual reduction in SR load and a reduction of calcium release amplitude induced by calcium release. This also explains why the long‐term effect of FGF23 on cardiomyocytes weakens myocardial contractility, which is mainly due to the remodelling of RyR2 caused by long‐term and persistent I_Ca,L_ entry. However, Navarro‐Garcia did not treat these cells for a long time. After only 1–3 min of treatment, he found that FGF23 could cause intracellular calcium disturbance. However, the high concentration of FGF23 he used indicated that the effect of FGF23 on cardiomyocytes may be concentration‐dependent. In this study, cardiomyocytes were interfered with their overexpression, and FGF23 protein produced by themselves could be enriched around the cells and act on cardiomyocytes in the form of autocrine or paracrine. However, the concentration of FGF23 generated by transcription is unpredictable, and the effect of FGF23 on cardiomyocytes is still limited by this method.

### 4.1 Limitations

FGF23 can be produced by cardiomyocytes, and non‐cardiomyocytes such as cardiac fibroblasts and endothelial cells can also express FGF23 protein. Up‐regulation of cardiac FGF23 in a cardiac remodelling environment may also be responsible for non‐cardiomyocytes. This study only investigated the mechanism of FGF23 and AF in cardiomyocytes, while not in other cells.

Gender differences play an important role in arrhythmia. However, this study exclusively involved male mice in animal experiments, leading to a lack of in‐depth discussion on gender differences when discussing the mechanism of cardiac FGF23 on the occurrence of atrial fibrillation.

The SERCA protein, as the primary calcium removal mechanism for cytosolic calcium, is regulated by phospholamban (PLN). This study only investigated SERCA protein expression and its functional alterations and did not explore the regulatory mechanisms of phospholamban on SERCA activity.

## 5 Conclusion

Cardio‐specific FGF23 knockout mitigates susceptibility to ISO‐induced atrial fibrillation. The vulnerability to atrial fibrillation is associated with a reduction in the occurrence of DAD, which arises from impaired NCX function. This alteration primarily stems from a decrease in intracellular calcium levels. Diminished membrane L‐type calcium current results in reduced calcium‐induced calcium release and decreased sarcoplasmic reticulum calcium load, ultimately leading to a decline in diastolic calcium ion leakage. These changes in calcium handling are correlated with the expression of Cav1.2 and RyR2, proteins involved in calcium transport regulation.

## Author Contributions

**Xiao‐Qian Li:** investigation (equal), writing – original draft (equal). **Mei‐Qiong Wu:** data curation (equal), investigation (equal), writing – review and editing (equal). **Li‐Hua Fang:** investigation (equal), writing – original draft (equal). **Qian Chen:** data curation (equal), investigation (equal). **Zhi‐Jie Chen:** data curation (equal), investigation (equal). **Zhu‐Hui Lin:** investigation (equal). **Jian‐Quan Chen:** data curation (equal). **Panashe Makota:** investigation (equal). **Yang Li:** conceptualization (equal), funding acquisition (equal), project administration (equal), writing – review and editing (equal). **Jian‐Cheng Zhang:** conceptualization (equal), funding acquisition (equal), project administration (equal).

## Ethics Statement

All our experimental procedures were approved by the Ethics Committee of PLA General Hospital and performed in accordance with the Guide for the Care and Use of Laboratory Animals published by the U.S. National Institutes of Health (Publication No. 23, revised 1996).

## Conflicts of Interest

The authors confirm that there are no conflicts of interest.

## Data Availability

The data that support the findings of this study are available from the corresponding author upon reasonable request.
